# A Review of the Immunologic Pathways Involved in Bullous Pemphigoid and Novel Therapeutic Targets

**DOI:** 10.3390/jcm11102856

**Published:** 2022-05-18

**Authors:** Mohsen Afarideh, Robert Borucki, Victoria P. Werth

**Affiliations:** 1 Corporal Michael J. Crescenz VA Medical Center, United States Department of Veterans Affairs, Philadelphia, PA 19104, USA; mohsen.afarideh@pennmedicine.upenn.edu (M.A.); robertbborucki@gmail.com (R.B.); 2 Department of Dermatology, Perelman School of Medicine, University of Pennsylvania, Philadelphia, PA 19104, USA

**Keywords:** bullous pemphigoid, immunology, therapeutic target, randomized controlled trials

## Abstract

Bullous pemphigoid (BP) is a rare, chronic antibody-mediated autoimmune blistering disease primarily affecting the elderly, with an age of onset over 60. Current treatment options are limited and involve the use of corticosteroids and immunosuppressants, but their long-term use is associated with significant morbidity and mortality. In Japan, human intravenous immunoglobin is approved for the treatment of corticosteroid-refractory BP. However, no treatment option is approved by the Food and Drug Administration for the management of BP. Therefore, developing effective therapies free of debilitating side effects is imperative. In this review, we summarize the main immunologic pathways involved in the pathogenesis of BP, with an emphasis on the role of eosinophils, immunoglobulins, cytokines such as the interleukin (IL)-4 and IL-5, and complements. We further discuss the latest advances with novel therapeutic targets tested for the management of BP. Ongoing efforts are needed to run well-designed controlled trials and test the efficacy and safety of investigational drugs while providing much-needed access to these medications for refractory patients who will not otherwise be able to afford them as off-label prescriptions.

## 1. Introduction

Bullous pemphigoid (BP) is a chronic antibody-mediated autoimmune blistering disease which occurs at an estimated annual incidence of 6–22 newly identified cases per million of the population, and primarily affects the elderly, with an age of onset over 60 [1,2,3,4,5]. Other significant risk factors for BP include a large group of drugs, as well as neurological/psychiatric comorbidities [6]. Drug-induced/associated BP is especially on the rise, with a variety of systemic agents among the common culprits. These include diuretics (e.g., furosemide, bumetanide, spironolactone), analgesics, d-penicillamine, antibiotics (e.g., amoxicillin, ciprofloxacin), psycholeptics (phenothiazine), potassium iodide, captopril, tumor necrosis factor (TNF) inhibitors, and the antidiabetic agents metformin and dipeptidyl peptidase-4 inhibitors (DPP-4is) such as vildagliptin, sildagliptin, saxagliptin, linagliptin, alogliptin [7]. Of note, long latency may suggest that certain BPs are drug-triggered (i.e., they initiate BP immunologically, but removing the drug does not help with controlling the disease). This is in contrast to true drug-induced skin disease, where removing the drug leads to the resolution of the disease [8].

Classic BP is characterized by tense subepidermal bullae, often along with eczematous or urticarial plaques and predominantly affects the trunk and flexural surfaces. However, atypical clinical variants still account for 20% of all patients, including only urticarial plaques or nonspecific skin findings [9]. The BP bullae are commonly preceded by a prodrome of pruritus [10], and this might be the sole BP presentation in select patients. Oral mucosa involvement is seen in up to one-fifth of the cases [11].

Current treatment methods are limited and rely on nonspecific anti-inflammatory and immunosuppressive therapies. The choice of treatment depends on the body surface area (BSA) involvement [6], with super-potent topical corticosteroids (40 g/day tapered over 12 months, or 10–30 g/day tapered over four months), which are equally effective but safer than systemic corticosteroids [12,13,14,15], and therefore recommended as the first-line for management of mild-to-moderate disease (up to 25% of total BSA involvement). Given the challenging practicality of daily ointment tube administration to deliver high doses of steroids [16], immunomodulator or non-immunosuppressant options such as doxycycline with or without niacinamide, represent an alternative option in select cases of localized, noninvasive BP [17]. Doxycycline has been proven to be non-inferior to prednisone for blister control in the short term, and safer in the long-term [17,18]. For severe BP (25–50% of total BSA involvement, or significant discomfort), systemic corticosteroids with a dose of 0.5 mg/kg/day (and up to 0.75 mg/kg/day in select refractory patients) is the treatment of choice [12]. The transition from induction to maintenance therapy typically takes up to four to six weeks of gradually tapered steroid doses. These treatments notably result in debilitating side effects, with significant morbidity/mortality in the elderly patients. The most effective adjuvant agents for refractory cases include mycophenolate mofetil, the combination of tetracycline and niacinamide, azathioprine, methotrexate, dapsone, and biologics such as rituximab, omalizumab, and dupilumab [19,20,21]. First-line combination therapy with rituximab and corticosteroids significantly improves complete remission rates along with reducing the cumulative dose of steroids without increasing the rate of complications [21]. Intravenous immunoglobin (IVIG), mainly comprised of IgG1 and IgG2, provides an alternative, safe and effective treatment for treatment-resistant BP patients. In Japan, human intravenous immunoglobin is the approved treatment for corticosteroid-refractory BP [22]. Rather surprisingly, no treatment is currently approved by the Food and Drug Administration (FDA) for BP.

## 2. Immunologic Pathways in BP

The pathogenesis of BP involves the combination of a predominantly humoral immune response against two well-established self-antigens, as well as some contributions from cellular immunity resulting in the characteristic tense blister formation [23]. The T-helper (Th)-2 pathway has been identified as the primary driver of antibody production in BP [24]. The self-antigens include BP antigen 180 (BP180, also called BPAG2 or type XVII collagen) and BP antigen 230 (BP230, also called epithelial isoform of BPAG1, BPAG1e). The presence of BP180-directed IgG is almost universal among patients with BP. In one study, it was found in 95% of patients with BP compared to the presence of BP230-targeted IgG in 70% of the same patient group [25]. IgG1 anti-BP180 was found as the most frequent subtype of IgG at 87%, and IgG4 anti-BP180 as the least frequent at 68% [25]. By comparison, IgE anti-BP180 and anti-BP230 antibodies were equally detected in only 22% of the same BP serum samples [25]. However, IgE antibodies may be detected in up to 77% of the cases [26]. The presence of IgA in addition to IgG auto-antibodies against BP180 (or less commonly, BP230) identifies the diagnosis of a mixed disease with features of both BP and linear immunoglobulin A bullous dermatosis (LABD). It has been suggested that the relative proportion of IgA and IgG antibodies correlate with the progression along one of the BP or LABD spectrums [27].

### 2.1. Role of IL-4

In parallel with the antibody response, the enhanced release of Th2-induced cytokines such as interleukin (IL)-4, IL-5, IL-6, IL-10, and IL-13 have been observed in the serum, skin biopsies, and blister fluid of patients with BP [28]. IL-4 is particularly essential in driving the Th2 response (i.e., autoantibody production) by switching B cell production to the IgE isotype and suppressing Th1 and Th17 differentiation [29]. One study showed that IL-4 has the highest association with BP of all cytokine responses [30]. Therefore, targeting IL-4 and its receptor (e.g., IL-4Rα subunit) has been proposed as theoretical drug targets for BP and other cutaneous autoimmune bullous diseases such as mucous membrane pemphigoid and pemphigus vulgaris [31].

### 2.2. Role of IL-5

IL-5 is the cytokine that promotes eosinophil growth. IL-5 functions as a major eosinophil chemoattractant and is important for their maturation and functional activity. Similar to IL-4, IL-5 is a Th2-induced cytokine detected in the skin lesions of patients with BP and characterizes the acute phase of BP, along with low levels of interferon-γ [32,33]. Th2 polarization may then in turn be perpetuated by the eosinophilic production of IL-4, IL-5, and IL-13 [34]. Furthermore, serum IL-5 has shown positive correlation with BP disease activity [35].

### 2.3. Role of IL-12/23

Elevated levels of IL-12 and IL-23 have been detected in blister fluids of BP patients [36]. In addition, IL-23 upregulates the secretion of the matrix metalloproteinase (MMP)-9, a major player of BP along the dermal-epidermal junction (DEJ) [36]. It has been shown that both IL-17 and IL-23 help with the identification of BP patients at risk of future relapse upon steroid therapy [36]. The ex vivo production of neutrophil-derived DNA extracellular traps, an important part of the pathogenesis of several autoimmune disorders, were stimulated during complementation of IL-23 during the relapse and post-treatment phases of BP [37]. Therefore, the uncontrollable inflammatory milieus secondary to the high levels of these molecules have prompted researchers to postulate their potential importance as novel biological targets for BP. Ustekinumab (anti-IL-12/23) is an FDA-approved agent for psoriasis, psoriatic arthritis and Crohn’s disease, and there have been recent studies looking into its potential efficacy for BP. However, there have been several concerning reports of paradoxical BP during ustekinumab therapy in psoriasis [38,39,40], necessitating careful studying and close dose monitoring implementation prior to manipulating the IL-12/23 pathway in BP in order to avoid disease exacerbation.

### 2.4. Role of IL-13

IL-13 is another crucial cytokine in the Th2 response, and shares many features with IL-4. Both cytokines are produced by Th2 cells, granulocytes, and monocytes/macrophages, and amplify IgE production by plasma cells [41,42]. They further share a common receptor subunit in IL-4Rα, and intracellular signaling molecules. Considering the ubiquitous presence of IL-4Rα in the body, every human cell has the potential to respond to IL-4 and IL-13 stimulatory signals [42]. Carrying a polymorphic genotype of the IL-13R C-allele (rs1800925) has been shown to protect against the development of BP, compared to the wild genotype of the same allele [28]. This suggests a potential to develop therapeutic strategies interfering specifically with the function of the IL-13 signaling pathway, an integral part in the pathogenesis of eosinophilic inflammatory disorders, including BP.

### 2.5. Role of IgE and Eosinophils

Anti-BP180 NC16A IgE and eosinophils can induce subepidermal blisters in mice [43]. However, this is not necessarily observed in humans. In patients, the ELISA-measured anti-BP180 NC16A IgE has been shown to correlate with BP clinical activity [44]. In a case-control setting, anti-BP180 NC16A serum IgE levels correlated with BP Disease Activity Index (BPDAI) scores, but both anti-BP180 NC16A IgE and IgG showed no association with urticarial or erythematous lesions [45]. However, the presence of anti-BP180 NC16A IgG correlated with the presence of erosions and blisters [45], consistent with the blocking of blister formation imposed by FcγR inhibition [43]. Nevertheless, the addition of anti-BP180 IgE to IgG anti-BP180 ELISA in the diagnostic assay increased diagnostic sensitivity by only 2.2% [45]. Considering the pathogenic role of anti-BP180 NC16A IgE in BP (correlation with BPDAI), its potential to guide therapy (e.g., to start omalizumab in BP patients with elevated IgE), and potential in helping to monitor response to treatment, its measurement may still be warranted in the management of BP.

Activated eosinophils (CD69+) provide another stimulus for BP pathogenesis, and their contribution is mediated via several different pathways [46]. Almost half of patients with BP have eosinophilia [47], with high serum levels of eosinophil cationic protein (ECP). Serum concentrations of ECP correlate with BP disease activity as well as IL-5 levels, a key cytokine linked with BP pathogenesis [48,49]. Other mechanisms include the role of proteinases, such as MMP-9, and neutrophil elastase in degrading BP180 and the subsequent cleavage of the DEJ [50]. TNF-α has been identified as a potent stimulator of pro-MMP-9 release [51]. Eosinophil granule proteins have also been detected in serum and blister fluids of BP patients [46]. Of note, they have been demonstrated in fully mature as well as early-stage and urticarial BP lesions, suggesting their importance as a potential marker of subclinical disease [52]. In addition, eosinophil production of network-like structures comprised of DNA, granule proteins, and nuclear proteins (i.e., eosinophil extracellular traps [EET]) have been suggested to accelerate the splitting of the DEJ [53]. Although this has been confirmed after reduced DEJ separation following the addition of DNase [54], the exact mechanism of EET-contributed DEJ separation remains unclear.

Eotaxin and monocyte chemoattractant protein (MCP)-4 are chemokines with significant roles in the selective recruitment of Th2 effector cells and eosinophils to BP-associated inflammatory sites, where they are detected in high levels within both tissue and blister fluids [55]. Eotaxin-1 and eotaxin-3 in particular, are highly upregulated in the serum and blister fluid of BP patients [56], and significantly associated with eosinophil numbers and activation [57]. In addition, eotaxin-1 is capable of binding to the CCR3 chemoreceptor expressed on eosinophils, resulting in eosinophil chemotaxis [57]. Therefore, eotaxin-1 and eotaxin-3 are implicated in BP development and their levels appear to be useful to monitor disease activity. Accordingly, their blockade could provide potentially novel treatment targets in BP [56,58].

### 2.6. Role of Complement

Various mediators of the complement immune system have been implicated in the etiology of BP. Elevated C5a and leukotriene B4 (LTB4) levels have been detected in the blister fluid of BP patients, and membrane attack complex (MAC, C5b9) was shown to be deposited along their DEJ [59,60,61,62]. Animal models with several deficient complement pathways (e.g., C3^−/−^, C4^−/−^, C5^−/−^, C5aR^−/−^) fail to develop characteristic BP lesions and blisters following injection with pathogenic autoantibodies [63,64,65,66]. Mutations in the C1q-binding sites of pathogenic BP autoantibodies renders them ineffective, suggesting the significant involvement of the classical complement pathway in BP [64]. It has been suggested that recombinant CD55/CD46 fusion protein is capable of inhibiting antibody-mediated complement activation at the outset of the classical complement pathway, providing new insights into the proposed complement-targeted treatment of BP [67]. On the other hand, cases of IgG4-dominant BP without C3 deposition have been reported, suggesting that IgG4 alone can mediate blister formation in such patients [68].

### 2.7. Drug-Related/Induced BP

There are a few key differences in terms of immunologic pathways involved in the pathogenesis of idiopathic BP compared to the drug-induced BP described in the literature. A higher proportion of non-inflammatory phenotypes (BPDAI: erythema/urticaria <10), low number of infiltrated lesional eosinophils and negative/low titers of anti-BP180 NC16A Ab has been found in Japanese patients with DPP-4i-induced BP who were mostly carrying the HLA-DQB1*03:01 allele [69,70]. By comparison, European studies have found no significant differences among these variables in the DPP-4i-induced BP and idiopathic BP [71].

Mechanistically, it has been shown that the inhibition of plasmin by DPP-4i reduces the degradation of the NC16A BP180 domain and may cause the breakdown of tolerance. DPP-4i has off-target effects on intracellular DPP-8 and DPP-9, whose inhibition activates NLRP1β and procaspase-1 [72].

## 3. Conventional Therapies

Among non-immunosuppressants, the combination of tetracyclines and niacinamide is particularly useful in the treatment of mild-to-moderate BP. Tetracyclines (dosed 0.5–2 g per day for tetracycline and 200–300 mg per day for doxycycline) dampen chemotaxis of neutrophils and eosinophils along with halting the enzymatic destruction caused by the MMPs. Niacinamide (dosed at 0.5–2.5 g per day) is thought to behave as an electron scavenger/phosphodiesterase inhibitor, ultimately stimulating the conversion of tryptophan to serotonin [73]. Dapsone is another non-immunosuppressant option shown to inhibit neutrophilic binding to the vascular endothelium, with the end result of decreased neutrophilic chemotaxis and activity by its enzyme, myeloperoxidase.

In the case of refractory BP, where treatment with topical or systemic steroids and/or conventional non-steroid immunosuppressants are ineffective, additional immunosuppressive therapy may be needed for steroid-sparing effects. These include mycophenolate mofetil, azathioprine, or methotrexate. Mycophenolate inhibits DNA synthesis of nucleotides and is used in a similar fashion to azathioprine for treating BP, with similar efficacy results, although with a better safety profile [74]. Azathioprine is a derivative of 6-mercaptopurine, with dual anti-inflammatory and immunosuppressive effects. Methotrexate is a cytotoxic folic acid analog resulting in the inhibition of the dihydrofolate reductase enzyme, and possesses additional anti-inflammatory properties [75]. It should be noted that all conventional therapies carry significant risks associated with their use, including but not limited to hepatotoxicity, myelosuppression, cardiopulmonary toxicities, gastrointestinal discomforts, oral ulcers, idiosyncratic hypersensitivity, and secondary malignancies [76].

## 4. Targeted Therapies

Dupilumab is a fully human monoclonal antibody that binds the IL-4Rα subunit. By binding this receptor, it blocks IL-4 and IL-13 signaling crucial to the type 2 inflammatory pathway. Dupilumab also inhibits the secretion of IL-31, a mediator of itch, by eosinophils. The medication was FDA-approved for atopic dermatitis in 2017 and is currently being studied in a number of diseases that involve the type 2 inflammatory pathway, including BP [77]. In 2018, the first successful case of dupilumab monotherapy for the treatment of refractory BP was reported [78]. Other case reports and series have shown promising results for the use of dupilumab in BP [78,79]. Abdat et al. retrospectively reviewed 13 patients between five sites treated with dupilumab. Seven patients achieved complete disease clearance and an additional five had disease control, with one patient not responding [80]. The medication was well tolerated with no adverse events (AEs) in this case series, with similar safety results published in dupilumab clinical trials and cohorts for other conditions, including in patients with moderate to severe disease [81]. Dupilumab relieves refractory itching not controlled by omalizumab [79]. There is currently a phase 2/3 randomized double-blind placebo-controlled trial for dupilumab use in BP underway (NCT04206553).

IL-5 is a proposed therapeutic target in BP, though there is limited published clinical data regarding related therapies. Mepolizumab is a humanized IgG1 kappa monoclonal antibody against IL-5 with FDA approval to treat asthma that was previously studied in BP [82]. In a phase 2 double-blind pilot study, mepolizumab failed to reach its primary endpoint in BP, and has not been investigated further. The high number of AEs were mostly related to the advanced age of patients and none were attributed to mepolizumab. This trial was limited by a small sample size (30 randomized subjects) and short duration of treatment (12 weeks) [83]. Benralizumab is another asthma medication with a similar therapeutic target, though this humanized IgG1 κ monoclonal antibody binds the IL-5Rα subunit on eosinophils and basophils, blocking their maturation and differentiation [84]. There have been no published studies in BP, though there is currently a phase 3 clinical trial underway (NCT04612790).

Omalizumab is a recombinant humanized IgG1 monoclonal antibody that blocks the binding of IgE to FcεRI receptors, and is currently FDA approved for asthma [85]. Because IgE autoantibodies are present in BP lesions, it is hypothesized that omalizumab interferes with the interactions of these autoantibodies with eosinophils and mast cells [85,86]. This treatment has been explored in case reports and series and was shown to be effective, particularly in patients with high IgE and eosinophil levels [85,87,88]. Yu et al. showed that five of six patients responded positively with no reported AE. Omalizumab was sufficient as a monotherapy in three of these patients [85]. Complete clearance was seen in six of 11 patients with refractory BP, and a partial response was seen in three others [89]. To date, no randomized placebo-controlled clinical trials have been conducted for omalizumab in BP. However, an open-label, single-group design, phase 3 clinical trial is currently underway to explore the safety and efficacy of omalizumab plus rituximab in the treatment of moderate-to-severe refractory BP (NCT04128176). In this trial, intravenous infusions of rituximab 1000 mg will be administered six months after the patient’s initial cycle of rituximab. In addition, omalizumab 300 mg will be injected subcutaneously every two weeks starting at baseline until week 52.

Another therapy studied in BP with some promise is bertilimumab, a fully human monoclonal antibody targeting eotaxin-1 (CCL-11). By binding this chemokine, the molecule impairs eosinophil migration to the skin, which is actively involved in BP lesions. A phase 2 open label study of nine patients met its primary and secondary endpoints in BP. Bertilimumab was well tolerated with a single serious AE unrelated to the drug. Limitations of this trial included lack of a control group, a small number of patients (*n* = 9), and a short duration of treatment (4 weeks) [90]. Following this study, the FDA granted Immune Pharmaceuticals fast track designation for bertilimumab to be studied in BP [91]. Further studies were never initiated as the company developing the molecule filed for bankruptcy in 2019 [92].

Ustekinumab is an IL-12 and IL-23 inhibitor that targets their shared p40 subunit and is currently FDA-approved for psoriasis, psoriatic arthritis, and Crohn’s disease. There is limited data available describing its role in BP, and the few cases published have mixed results. There are two case reports published of patients with bullous disease and psoriasis, one with BP and one with antilaminin-γ1 pemphigoid, reporting clearance of both diseases following treatment with ustekinumab [93,94]. Conversely, there are case reports in the literature that suggest ustekinumab as a culprit for drug-induced BP [38,40]. Despite these seemingly conflicting reports, there is currently an ongoing phase 2 open label study evaluating the efficacy and safety of ustekinumab as an adjuvant to the topical superpotent steroids in BP (NCT04117932).

Tildrakizumab, which targets the p19 subunit of IL-23, is FDA approved for the treatment of adult plaque psoriasis [95]. An open-label, single-arm phase 1 clinical trial (NCT04465292) is planning to enroll 16 patients to determine the efficacy of tildrakizumab for BP.

Sutimlimab (BIVV009, previously called TNT009) is a humanized IgG4 monoclonal antibody that inhibits C1s, a serine protease necessary for the dissemination of the classical complement pathway. By binding C1s, sutimlimab is hypothesized to disrupt the complement pathway involved in the chemoattraction of leukocytes in BP [96,97,98]. A first-in-human open-label phase 1 trial in 10 patients with active or past BP (NCT02502903) found that sutimlimab was both safe and tolerable, with common cold, fatigue, and headache being the most common AEs. Mild-to-moderate AEs comprised >97% of all AEs, although an 84-year-old patient died of cardiac decompensation. In this trial, sutimlimab inhibited the classical complement pathway in all patients. C3c deposition along the DEJ was partially or completely abrogated in four of five patients. Interestingly, C3c deposition recurred in three of four patients tested following washout of sutimlimab. [98]. Considering the promising results of the phase I study, FDA designated sutimlimab as an orphan drug for BP in 2017 to promote future clinical investigations [99]. A prospective, double-blind, placebo controlled, phase 1 clinical trial (NCT02502903) with a larger sample size continues enrollment.

Avdoralimab is a specific antibody against C5aR1, a component of the complement pathway previously shown to mediate pathogenic properties of anti-BP180 IgG. Conversely, C5aR2 has a protective effect against the development of BP [100]. An open-label, randomized, parallel group phase 2 clinical trial (NCT04563923) is expected to enroll 40 patients to evaluate the efficacy of avdoralimab as an adjuvant to the superpotent topical steroids for the treatment of BP.

Several ongoing studies are further investigating potential therapeutics capable of targeting dual prostaglandin and complement pathways. Data from KO mouse models and preclinical drug intervention programs support a causal role for C5a and LTB4 in the pathogenesis of BP [59,101,102]. In accordance with these observations, nomacopan (previously rVA576, coversin) is a second generation complement inhibitor which has activity against both C5, part of the MAC, and LTB4. In a recently completed phase 2 open label trial (NCT04035733), seven of nine patients responded to nomacopan, with no treatment-related AEs and results promising rapid control and minimizing the need for steroids in BP. The trial was limited by lack of a control group, the short duration of treatment (six weeks), inclusion of only mild-to-moderate BP at baseline (BPDAI of 10 to 56), and the exclusion of chronic, refractory BP patients [103]. Based on these results, the FDA granted a fast-track designation to nomacopan for the treatment of BP, and a phase 3 placebo-controlled trial is currently being planned [104]. This along with other targeted therapies are summarized in Table 1.

While some of the treatments covered here are available off-label for patients in need, others are still under development as investigational drugs and not widely accessible. Even the treatments available on the market are often cost-prohibitive if prescribed off-label. For this reason, clinical trials in BP are essential to increase access to treatments for these patients. Designing these trials presents a challenge as the disease is rare, and choosing appropriate endpoints can be difficult. Most trials are designed as add-on therapy vs. placebo in addition to oral corticosteroids, and it can be difficult to measure the response to a drug while attempting to standardize a corticosteroid taper in the background. These issues may have played a role in the failed mepolizumab trial, as 30 patients is a small number to see significant change, and the primary endpoint at 12 weeks may have been too short to see a difference with corticosteroids working in the background [83].

## 5. Conclusions

The growing understanding of BP on a cellular level has opened the door for the study of targeted therapies in this disease. Specifically, the roles of eosinophils and cytokines such as IL-4 and IL-5 have been discovered and explored as potential therapeutic targets. As currently available treatments generally involve corticosteroids and broad immunosuppression, there is potential that these newer therapies with specific molecular targets will have greater efficacy without such debilitating side effects. In addition to the development of new therapies, there is a large need for well-designed clinical trials to be conducted that will properly assess the efficacy of these therapies. These new therapies, along with their controlled study in clinical trials, will be essential in providing patients with relief for this debilitating disease.

## Figures and Tables

**Table 1:** Targeted therapies currently under investigation for use in BP.

Therapeutic Target	Medication	Phase of Study
IL4	Dupilumab	Phase 2/3 underway
IgE	Omalizumab	Previous case series
IL5	Mepolizumab	Failed phase 2 completed
	Benralizumab	Phase 3 underway
Eotaxin-1	Bertilimumab	Positive phase 2 completed
IL12/IL23	Ustekinumab	Phase 2 underway
IL-23	Tildrakizumab	Phase 1 underway
C1s	Sutimlimab	Positive phase 1 completed
C5aR1	Avdoralimab	Phase 2 underway
C5/LTB4	Nomacopan	Positive phase 2 completed

## References

[1] Amber K.T., Murrell D.F., Schmidt E., Joly P., Borradori L. (2018). Autoimmune subepidermal bullous diseases of the skin and mucosae: Clinical features, diagnosis, and management. Clin. Rev. Allergy Immunol..

[2] Serwin A.B., Musialkowska E., Piascik M. (2014). Incidence and mortality of bullous pemphigoid in north-east Poland (Podlaskie Province), 1999–2012: A retrospective bicentric cohort study. Int. J. Dermatol..

[3] Försti A.K., Jokelainen J., Timonen M., Tasanen K. (2014). Increasing incidence of bullous pemphigoid in Northern Finland: A retrospective database study in Oulu University Hospital. Br. J. Dermatol..

[4] Wertenteil S., Garg A., Strunk A., Alloo A. (2019). Prevalence estimates for pemphigoid in the United States: A sex-adjusted and age-adjusted population analysis. J. Am. Acad. Dermatol..

[5] Joly P., Baricault S., Sparsa A., Bernard P., Bédane C., Duvert-Lehembre S., Courville P., Bravard P., Rémond B., Doffoel-Hantz V. (2012). Incidence and mortality of bullous pemphigoid in France. J. Investig. Dermatol..

[6] Di Lernia V., Casanova D.M., Goldust M., Ricci C. (2020). Pemphigus vulgaris and bullous pemphigoid: Update on diagnosis and treatment. Dermatol. Pract. Concept..

[7] Fania L., Salemme A., Provini A., Pagnanelli G., Collina M.C., Abeni D., Didona B., Di Zenzo G., Mazzanti C. (2018). Detection and characterization of IgG, IgE, and IgA autoantibodies in patients with bullous pemphigoid associated with dipeptidyl peptidase-4 inhibitors. J. Am. Acad. Dermatol..

[8] Nishie W., Tasanen K. (2019). Gliptin-associated bullous pemphigoid: A valuable model of the mechanism of breakdown of immune tolerance against BP180. J. Investig. Dermatol..

[9] Schmidt T., Sitaru C., Amber K., Hertl M. (2014). BP180-and BP230-specific IgG autoantibodies in pruritic disorders of the elderly: A preclinical stage of bullous pemphigoid?. Br. J. Dermatol..

[10] Schmidt E., Zillikens D. (2013). Pemphigoid diseases. Lancet.

[11] Della Torre R., Combescure C., Cortés B., Marazza G., Beltraminelli H., Naldi L., Borradori L. (2012). Clinical presentation and diagnostic delay in bullous pemphigoid: A prospective nationwide cohort. Br. J. Dermatol..

[12] Feliciani C., Joly P., Jonkman M.F., Zambruno G., Zillikens D., Ioannides D., Kowalewski C., Jedlickova H., Kárpáti S., Marinovic B. (2015). Management of bullous pemphigoid: The European Dermatology Forum consensus in collaboration with the European Academy of Dermatology and Venereology. Br. J. Dermatol..

[13] Kirtschig G., Middleton P., Bennett C., Murrell D., Wojnarowska F., Khumalo N. (2011). Interventions for bullous pemphigoid: A summarised Cochrane review. Clin. Exp. Dermatol..

[14] Joly P., Roujeau J.-C., Benichou J., Picard C., Dreno B., Delaporte E., Vaillant L., D’Incan M., Plantin P., Bedane C. (2002). A comparison of oral and topical corticosteroids in patients with bullous pemphigoid. N. Engl. J. Med..

[15] Joly P., Roujeau J.-C., Benichou J., Delaporte E., D’incan M., Dreno B., Bedane C., Sparsa A., Gorin I., Picard C. (2009). A comparison of two regimens of topical corticosteroids in the treatment of patients with bullous pemphigoid: A multicenter randomized study. J. Investig. Dermatol..

[16] Eming R., Sticherling M., Hofmann S.C., Hunzelmann N., Kern J.S., Kramer H., Pfeiffer C., Schuster V., Zillikens D., Goebeler M. (2015). S2k guidelines for the treatment of pemphigus vulgaris/foliaceus and bullous pemphigoid. J. Der Dtsch. Dermatol. Ges. J. Ger. Soc. Dermatol. JDDG.

[17] Williams H.C., Wojnarowska F., Kirtschig G., Mason J., Godec T.R., Schmidt E., Chalmers J.R., Childs M., Walton S., Harman K. (2017). Doxycycline versus prednisolone as an initial treatment strategy for bullous pemphigoid: A pragmatic, non-inferiority, randomised controlled trial. Lancet.

[18] Yamagami J. (2018). Recent advances in the understanding and treatment of pemphigus and pemphigoid. F1000Research.

[19] Sticherling M., Franke A., Aberer E., Glaeser R., Hertl M., Pfeiffer C., Rzany B., Schneider S., Shimanovich I., Werfel T. (2017). An open, multicentre, randomized clinical study in patients with bullous pemphigoid comparing methylprednisolone and azathioprine with methylprednisolone and dapsone. Br. J. Dermatol..

[20] Kremer N., Snast I., Cohen E.S., Hodak E., Mimouni D., Lapidoth M., Mazor S., Levi A. (2019). Rituximab and omalizumab for the treatment of bullous pemphigoid: A systematic review of the literature. Am. J. Clin. Dermatol..

[21] Zhou T., Peng B., Geng S. (2021). Emerging Biomarkers and Therapeutic Strategies for Refractory Bullous Pemphigoid. Front. Immunol..

[22] Amagai M., Ikeda S., Hashimoto T., Mizuashi M., Fujisawa A., Ihn H., Matsuzaki Y., Ohtsuka M., Fujiwara H., Furuta J. (2017). A randomized double-blind trial of intravenous immunoglobulin for bullous pemphigoid. J. Dermatol. Sci..

[23] Bernard P., Antonicelli F. (2017). Bullous pemphigoid: A review of its diagnosis, associations and treatment. Am. J. Clin. Dermatol..

[24] Fang H., Li Q., Wang G. (2020). The role of T cells in pemphigus vulgaris and bullous pemphigoid. Autoimmun. Rev..

[25] Iwata Y., Komura K., Kodera M., Usuda T., Yokoyama Y., Hara T., Muroi E., Ogawa F., Takenaka M., Sato S. (2008). Correlation of IgE autoantibody to BP180 with a severe form of bullous pemphigoid. Arch. Dermatol..

[26] Messingham K.N., Crowe T.P., Fairley J.A. (2019). The intersection of IgE autoantibodies and eosinophilia in the pathogenesis of bullous pemphigoid. Front. Immunol..

[27] Mohanan S., Criton S., Abraham U.M. (2020). Mixed immunobullous disease in infants: Falls in bullous pemphigoid-linear IgA spectrum?. Indian J. Paediatr. Dermatol..

[28] Tabatabaei-Panah P.-S., Moravvej H., Alirajab M., Etaaty A., Geranmayeh M., Hosseine F., Khansari A., Mahdian M., Mirhashemi M., Parvizi S. (2020). Association between TH2 Cytokine Gene Polymorphisms and Risk of Bullous Pemphigoid. Immunol. Investig..

[29] Zhang J., Fang H., Shen S., Dang E., Li Q., Qiao P., Qiao H., Wang G. (2018). Identification of Immunodominant Th2-Cell Epitopes in Chinese Patients with Bullous Pemphigoid. J. Investig. Dermatol..

[30] Pickford W.J., Gudi V., Haggart A.M., Lewis B.J., Herriot R., Barker R.N., Ormerod A.D. (2015). T cell participation in autoreactivity to NC16a epitopes in bullous pemphigoid. Clin. Exp. Immunol..

[31] Russo R., Cozzani E., Gasparini G., Parodi A. (2020). Targeting interleukin 4 receptor α: A new approach to the treatment of cutaneous autoimmune bullous diseases?. Dermatol. Ther..

[32] Giomi B., Caproni M., Calzolari A., Bianchi B., Fabbri P. (2002). Th1, Th2 and Th3 cytokines in the pathogenesis of bullous pemphigoid. J. Dermatol. Sci..

[33] Feliciani C., Toto P., Mohammad Pour S., Coscione G., Amerio P. (1999). A Th2-like cytokine response is involved in bullous pemphigoid. the role of IL-4 and IL-5 in the pathogenesis of the disease. Int. J. Immunopathol. Pharmacol..

[34] Spencer L.A., Szela C.T., Perez S.A., Kirchhoffer C.L., Neves J.S., Radke A.L., Weller P.F. (2009). Human eosinophils constitutively express multiple Th1, Th2, and immunoregulatory cytokines that are secreted rapidly and differentially. J. Leukoc. Biol..

[35] D’auria L., Pietravalle M., Mastroianni A., Ferraro C., Mussi A., Bonifati C., Giacalone B., Ameglio F. (1998). IL-5 levels in the serum and blister fluid of patients with bullous pemphigoid: Correlations with eosinophil cationic protein, RANTES, IgE and disease severity. Arch. Dermatol. Res..

[36] Plée J., Le Jan S., Giustiniani J., Barbe C., Joly P., Bedane C., Vabres P., Truchetet F., Aubin F., Antonicelli F. (2015). Integrating longitudinal serum IL-17 and IL-23 follow-up, along with autoantibodies variation, contributes to predict bullous pemphigoid outcome. Sci. Rep..

[37] Giusti D., Bini E., Terryn C., Didier K., Le Jan S., Gatouillat G., Durlach A., Nesmond S., Muller C., Bernard P. (2019). NET formation in bullous pemphigoid patients with relapse is modulated by IL-17 and IL-23 interplay. Front. Immunol..

[38] Marin M., Alzueta N., Castresana M., Gascón A., Pío M. (2021). Bullous pemphigoid induced by ustekinumab: A case report. Eur. J. Hosp. Pharm..

[39] Onsun N., Sallahoglu K., Dizman D., Su Ö., Tosuner Z. (2017). Bullous pemphigoid during ustekinumab therapy in a psoriatic patient. Eur. J. Dermatol..

[40] Le Guern A., Alkeraye S., Vermersch-Langlin A., Coupe P., Vonarx M. (2015). Bullous pemphigoid during ustekinumab therapy. JAAD Case Rep..

[41] Moyle M., Cevikbas F., Harden J.L., Guttman-Yassky E. (2019). Understanding the immune landscape in atopic dermatitis: The era of biologics and emerging therapeutic approaches. Exp. Dermatol..

[42] McCormick S.M., Heller N.M. (2015). Commentary: IL-4 and IL-13 receptors and signaling. Cytokine.

[43] Lin L., Hwang B.J., Culton D.A., Li N., Burette S., Koller B.H., Messingham K.A., Fairley J.A., Lee J.J., Hall R.P. (2018). Eosinophils Mediate Tissue Injury in the Autoimmune Skin Disease Bullous Pemphigoid. J. Investig. Dermatol..

[44] Kalowska M., Ciepiela O., Kowalewski C., Demkow U., Schwartz R.A., Wozniak K. (2016). Enzyme-linked Immunoassay Index for Anti-NC16a IgG and IgE Auto-antibodies Correlates with Severity and Activity of Bullous Pemphigoid. Acta Derm. Venereol..

[45] Van Beek N., Lüttmann N., Huebner F., Recke A., Karl I., Schulze F.S., Zillikens D., Schmidt E. (2017). Correlation of serum levels of IgE autoantibodies against BP180 with bullous pemphigoid disease activity. JAMA Dermatol..

[46] Amber K.T., Valdebran M., Kridin K., Grando S.A. (2018). The role of eosinophils in bullous pemphigoid: A developing model of eosinophil pathogenicity in mucocutaneous disease. Front. Med..

[47] Kridin K. (2018). Peripheral eosinophilia in bullous pemphigoid: Prevalence and influence on the clinical manifestation. Br. J. Dermatol..

[48] Giusti D., Gatouillat G., Le Jan S., Plée J., Bernard P., Antonicelli F., Pham B.N. (2017). Eosinophil Cationic Protein (ECP), a predictive marker of bullous pemphigoid severity and outcome. Sci. Rep..

[49] Yamashita C., Nakamizo S., Honda Y., Dainichi T., Kabashima K. (2017). Combination therapy of prednisolone and iv immunoglobulin treatment decreases circulating interleukin-5 and eosinophils in a patient with bullous pemphigoid. J. Dermatol..

[50] Verraes S., Hornebeck W., Bernard P., Polette M., Borradori L. (2001). Respective contribution of neutrophil elastase and matrix metalloproteinase 9 in the degradation of BP180 (type XVII collagen) in human bullous pemphigoid. J. Investig. Dermatol..

[51] Wiehler S., Cuvelier S.L., Chakrabarti S., Patel K.D. (2004). p38 MAP kinase regulates rapid matrix metalloproteinase-9 release from eosinophils. Biochem. Biophys. Res. Commun..

[52] Borrego L., Maynard B., Peterson E.A., George T., Iglesias L., Peters M.S., Newman W., Gleich G.J., Leiferman K.M. (1996). Deposition of eosinophil granule proteins precedes blister formation in bullous pemphigoid. Comparison with neutrophil and mast cell granule proteins. Am. J. Pathol..

[53] Cortjens B., van Woensel J., Bem R. (2017). Neutrophil extracellular traps in respiratory disease: Guided anti-microbial traps or toxic webs?. Paediatr. Respir. Rev..

[54] Simon D., Hoesli S., Roth N., Staedler S., Yousefi S., Simon H.-U. (2011). Eosinophil extracellular DNA traps in skin diseases. J. Allergy Clin. Immunol..

[55] Abdelilah S.G., Wellemans V., Agouli M., Guenounou M., Hamid Q., Beck L.A., Lamkhioued B. (2006). Increased expression of Th2-associated chemokines in bullous pemphigoid disease. Role of eosinophils in the production and release of these chemokines. Clin. Immunol..

[56] Liu Y., Wang Y., Chen X., Jin H., Li L. (2020). Factors associated with the activity and severity of bullous pemphigoid: A review. Ann. Med..

[57] Günther C., Wozel G., Meurer M., Pfeiffer C. (2011). Up-regulation of CCL11 and CCL26 is associated with activated eosinophils in bullous pemphigoid. Clin. Exp. Immunol..

[58] Lee J., Werth V.P., Hall R.P., Eming R., Fairley J.A., Fajgenbaum D.C., Harman K.E., Jonkman M.F., Korman N.J., Ludwig R.J. (2018). Perspective from the 5th international pemphigus and pemphigoid foundation scientific conference. Front. Med..

[59] Sezin T., Murthy S., Attah C., Seutter M., Holtsche M.M., Hammers C.M., Schmidt E., Meshrkey F., Mousavi S., Zillikens D. (2019). Dual inhibition of complement factor 5 and leukotriene B4 synergistically suppresses murine pemphigoid disease. JCI Insight.

[60] Kawana S., Ueno A., Nishiyama S. (1990). Increased levels of immunoreactive leukotriene B4 in blister fluids of bullous pemphigoid patients and effects of a selective 5-lipoxygenase inhibitor on experimental skin lesions. Acta Derm.Venereol..

[61] Diaz-Perez J.L., Jordon R.E. (1976). The complement system in bullous pemphigoid: IV. Chemotactic activity in blister fluid. Clin. Immunol. Immunopathol..

[62] Dahl M.V., Falk R.J., Carpenter R., Michael A.F. (1984). Deposition of the membrane attack complex of complement in bullous pemphigoid. J. Investig. Dermatol..

[63] Heimbach L., Li Z., Berkowitz P., Zhao M., Li N., Rubenstein D.S., Diaz L.A., Liu Z. (2011). The C5a receptor on mast cells is critical for the autoimmune skin-blistering disease bullous pemphigoid. J. Biol. Chem..

[64] Li Q., Ujiie H., Shibaki A., Wang G., Moriuchi R., Qiao H.-J., Morioka H., Shinkuma S., Natsuga K., Long H.A. (2010). Human IgG1 monoclonal antibody against human collagen 17 noncollagenous 16A domain induces blisters via complement activation in experimental bullous pemphigoid model. J. Immunol..

[65] Yamamoto K., Inoue N., Masuda R., Fujimori A., Saito T., Imajoh-Ohmi S., Shinkai H., Sakiyama H. (2002). Cloning of hamster type XVII collagen cDNA, and pathogenesis of anti-type XVII collagen antibody and complement in hamster bullous pemphigoid. J. Investig. Dermatol..

[66] Liu Z., Giudice G.J., Swartz S.J., Fairley J.A., Till G.O., Troy J.L., Diaz L.A. (1995). The role of complement in experimental bullous pemphigoid. J. Clin. Investig..

[67] Qiao P., Luo Y.-X., Zhi D.-L., Wang G., Dang E.-L. (2021). Blockade of complement activation in bullous pemphigoid by using recombinant CD55-CD46 fusion protein. Chin. Med. J..

[68] Dainichi T., Nishie W., Yamagami Y., Sonobe H., Ujiie H., Kaku Y., Kabashima K. (2016). Bullous pemphigoid suggestive of complement-independent blister formation with anti-BP 180 IgG4 autoantibodies. Br. J. Dermatol..

[69] Izumi K., Nishie W., Mai Y., Wada M., Natsuga K., Ujiie H., Iwata H., Yamagami J., Shimizu H. (2016). Autoantibody profile differentiates between inflammatory and noninflammatory bullous pemphigoid. J. Investig. Dermatol..

[70] Ujiie H., Muramatsu K., Mushiroda T., Ozeki T., Miyoshi H., Iwata H., Nakamura A., Nomoto H., Cho K.Y., Sato N. (2018). HLA-DQB1* 03: 01 as a biomarker for genetic susceptibility to bullous pemphigoid induced by DPP-4 inhibitors. J. Investig. Dermatol..

[71] Bellinato F., Maurelli M., Schena D., Gisondi P., Girolomoni G. (2020). Clinical and immunological profile of patients with dipeptidyl peptidase-4 inhibitor-associated bullous pemphigoid. Ital. J. Dermatol. Venerol..

[72] Lankas G.R., Leiting B., Roy R.S., Eiermann G.J., Beconi M.G., Biftu T., Chan C.-C., Edmondson S., Feeney W.P., He H. (2005). Dipeptidyl peptidase IV inhibition for the treatment of type 2 diabetes: Potential importance of selectivity over dipeptidyl peptidases 8 and 9. Diabetes.

[73] Shimoyama M., Kawai M., Nasu S., Shioji K., Hoshi Y. (1975). Inhibition of adenosine 3′, 5′-monophosphate phosphodiesterase by nicotinamide and its homologues in vitro. Physiol. Chem. Phys..

[74] Beissert S., Werfel T., Frieling U., Böhm M., Sticherling M., Stadler R., Zillikens D., Rzany B., Hunzelmann N., Meurer M. (2007). A comparison of oral methylprednisolone plus azathioprine or mycophenolate mofetil for the treatment of bullous pemphigoid. Arch. Dermatol..

[75] Kirtschig G., Khumalo N.P. (2004). Management of bullous pemphigoid. Am. J. Clin. Dermatol..

[76] Khalid S.N., Khan Z.A., Ali M.H., Almas T., Khedro T., Nagarajan V.R. (2021). A blistering new era for bullous pemphigoid: A scoping review of current therapies, ongoing clinical trials, and future directions. Ann. Med. Surg..

[77] Simpson E.L., Akinlade B., Ardeleanu M. (2017). Two Phase 3 Trials of Dupilumab versus Placebo in Atopic Dermatitis. N. Engl. J. Med..

[78] Kaye A., Gordon S.C., Deverapalli S.C., Her M.J., Rosmarin D. (2018). Dupilumab for the treatment of recalcitrant bullous pemphigoid. JAMA Dermatol..

[79] Seyed Jafari S.M., Feldmeyer L., Bossart S., Simon D., Schlapbach C., Borradori L. (2020). Case Report: Combination of Omalizumab and Dupilumab for Recalcitrant Bullous Pemphigoid. Front. Immunol..

[80] Abdat R., Waldman R.A., de Bedout V., Czernik A., Mcleod M., King B., Gordon S., Ahmed R., Nichols A., Rothe M. (2020). Dupilumab as a novel therapy for bullous pemphigoid: A multicenter case series. J. Am. Acad. Dermatol..

[81] Fargnoli M., Esposito M., Ferrucci S., Girolomoni G., Offidani A., Patrizi A., Peris K., Costanzo A., Malara G., Pellacani G. (2019). Real-life experience on effectiveness and safety of dupilumab in adult patients with moderate-to-severe atopic dermatitis. J. Dermatol. Treat..

[82] Pavord I.D., Korn S., Howarth P., Bleecker E.R., Buhl R., Keene O.N., Ortega H., Chanez P. (2012). Mepolizumab for severe eosinophilic asthma (DREAM): A multicentre, double-blind, placebo-controlled trial. Lancet.

[83] Simon D., Yousefi S., Cazzaniga S., Bürgler C., Radonjic S., Houriet C., Heidemeyer K., Klötgen H.W., Kozlowski E., Borradori L. (2020). Mepolizumab failed to affect bullous pemphigoid: A randomized, placebo-controlled, double-blind phase 2 pilot study. Allergy.

[84] FitzGerald J.M., Bleecker E.R., Nair P., Korn S., Ohta K., Lommatzsch M., Ferguson G.T., Busse W.W., Barker P., Sproule S. (2016). Benralizumab, an anti-interleukin-5 receptor α monoclonal antibody, as add-on treatment for patients with severe, uncontrolled, eosinophilic asthma (CALIMA): A randomised, double-blind, placebo-controlled phase 3 trial. Lancet.

[85] Yu K.K., Crew A.B., Messingham K.A., Fairley J.A., Woodley D.T. (2014). Omalizumab therapy for bullous pemphigoid. J. Am. Acad. Dermatol..

[86] Fairley J.A., Burnett C.T., Fu C.L., Larson D.L., Fleming M.G., Giudice G.J. (2007). A pathogenic role for IgE in autoimmunity: Bullous pemphigoid IgE reproduces the early phase of lesion development in human skin grafted to nu/nu mice. J. Investig. Dermatol..

[87] Fairley J.A., Baum C.L., Brandt D.S., Messingham K.A. (2009). Pathogenicity of IgE in autoimmunity: Successful treatment of bullous pemphigoid with omalizumab. J. Allergy Clin. Immunol..

[88] London V.A., Kim G.H., Fairley J.A., Woodley D.T. (2012). Successful treatment of bullous pemphigoid with omalizumab. Arch. Dermatol..

[89] Lonowski S., Sachsman S., Patel N., Truong A., Holland V. (2020). Increasing evidence for omalizumab in the treatment of bullous pemphigoid. JAAD Case Rep..

[90] Fiorino A.S., Baum S., Czernik A., Hall R., Zeeli T., Baniel A., Sinha A.A., Seiffert-Sinha K., Kolatch B., Zhang Z. (2019). 570 Safety and efficacy of bertilimumab, a human anti-eotaxin-1 monoclonal antibody, in bullous pemphigoid in a phase 2a study. J. Investig. Dermatol..

[91] Rossi K. FDA Grants Fast Track Designation to Bullous Pemphigoid Treatment, Bertilimumab. https://www.hcplive.com/view/fda-grants-fast-track-designation-bullous-pemphigoid-treatment-bertilimumab.

[92] Pharmaceuticals I. (2019). Immune Pharmaceuticals Files for Chapter 11 Protection. https://www.globenewswire.com/news-release/2019/02/19/1734168/0/en/Immune-Pharmaceuticals-Files-for-Chapter-11-Protection.html.

[93] Majima Y., Yagi H., Tateishi C., Groth S., Schmidt E., Zillikens D., Koga H., Hashimoto T., Tokura Y. (2013). A successful treatment with ustekinumab in a case of antilaminin-γ1 pemphigoid associated with psoriasis. Br. J. Dermatol..

[94] Loget J., Plée J., Antonicelli F., Bernard P. (2017). A successful treatment with ustekinumab in a case of relapsing bullous pemphigoid associated with psoriasis. J. Eur. Acad. Dermatol. Venereol..

[95] Armstrong A.W., Read C. (2020). Pathophysiology, clinical presentation, and treatment of psoriasis: A review. JAMA.

[96] Nikitin P.A., Rose E.L., Byun T.S., Parry G.C., Panicker S. (2019). C1s Inhibition by BIVV009 (Sutimlimab) Prevents Complement-Enhanced Activation of Autoimmune Human B Cells In Vitro. J. Immunol..

[97] Bartko J., Schoergenhofer C., Schwameis M., Firbas C., Beliveau M., Chang C., Marier J.F., Nix D., Gilbert J.C., Panicker S. (2018). A Randomized, First-in-Human, Healthy Volunteer Trial of sutimlimab, a Humanized Antibody for the Specific Inhibition of the Classical Complement Pathway. Clin. Pharmacol. Ther..

[98] Freire P.C., Muñoz C.H., Derhaschnig U., Schoergenhofer C., Firbas C., Parry G.C., Panicker S., Gilbert J.C., Stingl G., Jilma B. (2019). Specific inhibition of the classical complement pathway prevents C3 deposition along the dermal-epidermal junction in bullous pemphigoid. J. Investig. Dermatol..

[99] Kushner C.J., Payne A.S. (2018). Increasing the complement of therapeutic options in bullous pemphigoid. J. Investig. Dermatol..

[100] Karsten C.M., Beckmann T., Holtsche M.M., Tillmann J., Tofern S., Schulze F.S., Heppe E.N., Ludwig R.J., Zillikens D., König I.R. (2018). Tissue destruction in bullous pemphigoid can be complement independent and may be mitigated by C5aR2. Front. Immunol..

[101] Sezin T., Krajewski M., Wutkowski A., Mousavi S., Chakievska L., Bieber K., Ludwig R.J., Dahlke M., Rades D., Schulze F.S. (2017). The leukotriene B4 and its receptor BLT1 act as critical drivers of neutrophil recruitment in murine bullous pemphigoid-like epidermolysis bullosa acquisita. J. Investig. Dermatol..

[102] Edwards G., Diercks G.F., Seelen M.A., Horvath B., Van Doorn M., Damman J. (2019). Complement activation in autoimmune bullous dermatoses: A comprehensive review. Front. Immunol..

[103] Nunn M., Fettiplace J., Khindri S. (2021). Disease Remission During a Short-term Treatment Phase II Study of Nomacopan in Mild-to-moderate Bullous Pemphigoid—With Final Plan for Phase III Trial. J. Am. Acad. Dermatol..

[104] Plc A.T. (2021). Akari Therapeutics Receives FDA Fast Track Designation for Nomacopan for the Treatment of Bullous Pemphigoid. https://www.globenewswire.com/news-release/2021/04/28/2218574/0/en/Akari-Therapeutics-Receives-FDA-Fast-Track-Designation-for-Nomacopan-for-the-Treatment-of-Bullous-Pemphigoid.html.

